# Hair fragility (trichorrhexis nodosa) in alopecic Pomeranian dogs

**DOI:** 10.1111/vde.13296

**Published:** 2024-09-30

**Authors:** Erin Brennan, Jonna Juhola, Ewan A. Ferguson, Anette Loeffler, Rosario Cerundolo, Anke Hendricks, Ross Bond

**Affiliations:** ^1^ Department of Clinical Sciences and Services Royal Veterinary College Hatfield Herts UK; ^2^ Bayswater Referral Clinic Bayswater, London UK

**Keywords:** alopecia, hair cycle arrest, hair fragility, Pomeranian, trichorrhexis nodosa

## Abstract

**Background:**

While alopecia associated with hair cycle arrest (HCA, Alopecia X) is well‐recognised in Pomeranian dogs, the authors are unaware of reports of concurrent hair fragility.

**Hypothesis/Objectives:**

Following the observation of frequent hair shaft abnormalities in alopecic Pomeranians, we hypothesised that hair fragility events would be more frequent in dogs with a phenotype of HCA when compared to dogs with normal coats.

**Animals:**

Eight alopecic Pomeranian dogs or crosses with a phenotype of HCA and 36 unaffected Pomeranians with owner‐reported normal hair coats.

**Materials and Methods:**

All affected dogs underwent dermatological examination and clinicopathological evaluations. Hair samples, plucked from affected areas or obtained by brushing from unaffected dogs, were examined microscopically for structural abnormalities. Hair fragility events were characterised as trichorrhexis nodosa (TN) or longitudinal splits and were expressed per 10 mg of hair. A reference interval was calculated from the number of hair fragility events in the samples from unaffected dogs.

**Results:**

The upper reference limit (with 90% confidence) from samples of 35 unaffected Pomeranians (one outlier excluded) was 9.75 hair fragility events per 10 mg of hair. The median (range) of fragility events in eight dogs with a phenotype of HCA was 66.0 (30.2–166.7) per 10 mg of hair.

**Conclusions and Clinical Relevance:**

Clinicians should routinely perform trichography in Pomeranians with HCA because abundant hair abnormalities, particularly TN, may contribute to poor hair coat quality. Further studies are required to establish the pathophysiology of and treatments for these fragility events and to determine their predictive value for HCA.

## Graphical abstract

**Background** – Alopecia associated with hair cycle arrest (HCA, Alopecia X) is well‐recognised in Pomeranian dogs. The authors are unaware of reports of hair fragility in affected dogs. **Hypothesis/Objectives** – Following the observation of frequent hair shaft abnormalities in alopecic Pomeranians, we hypothesised that hair fragility events would be more frequent in dogs with a phenotype of HCA when compared to dogs with normal coats. **Conclusions and Clinical Relevance** – Clinicians should routinely perform trichography in Pomeranians with HCA because abundant hair abnormalities, particularly trichorrhexis nodosa, may contribute to poor hair coat quality. Further studies are required to establish the pathophysiology of and treatments for these fragility events and to determine their predictive value for HCA.

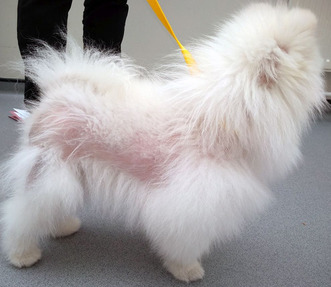

## Zusammenfassung

**Hintergrund:**

Eine Alopezie, die mit einem Haarzyklus‐Stop (HCA, Alopecia X) assoziiert wird, ist beim Pomeranian (Zwergspitz) gut bekannt. Nach bestem Wissen der Autoren gibt es keine Publikationen über die Brüchigkeit der Haare bei den betroffenen Hunden.

**Hypothese/Ziele:**

Nach der häufigen Beobachtung einer Haarschaft Abnormalität bei haarlosen Pomeranian, hypothetisierten wir, dass die Haarfragilität häufiger bei Hunden mit dem Phänotyp einer HCA im Vergleich zu Hunden mit normalem Haarkleid auftreten würde.

**Tiere:**

Acht Pomeranian oder ihre Kreuzungen mit Alopezie und einem Phänotyp von HCA und 36 nicht betroffene Pomeranian mit von den BesitzerInnen berichtetem normalem Haarkleid.

**Materialien und Methoden:**

Alle betroffenen Hunde wurden dermatologisch untersucht und klinisch‐pathologisch evaluiert. Haarproben, gezupft von den betroffenen Stellen oder gebürstet von nicht betroffenen Hunden wurden mikroskopisch auf strukturelle Abnormalitäten untersucht. Haarbrüchigkeit war charakterisiert durch eine Trichorrhexis nodosa (TN) oder longitudinale Aufsplitterung und wurde pro 10 mg Haare erfasst. Ein Referenzintervall wurde aus der Anzahl der auftretenden Haarbrüchigkeit der Proben von nicht betroffenen Hunden kalkuliert.

**Ergebnisse:**

Das obere Referenzlimit (mit 90% Konfidenz) von Proben der 35 nicht betroffenen Pomeranian (ein Ausreißer inkludiert) betrug 9,75 Haarbrüchigkeit pro 10 mg Haare. Der Medianwert der Haarbrüchigkeit der acht Hunde mit einem Phänotyp von HCA betrug 66,0 (30,2‐166,7) pro 10 mg Haar.

**Schlussfolgerungen und klinische Bedeutung:**

KlinikerInnen sollten routinemäßig ein Trichogramm bei Pomeranian mit HCA durchführen, da ausgedehnte Abnormalitäten, vor allem die Trichorrhexis nodosa, zu einer schlechten Haarkleidqualität beitragen könnten. Es sind weitere Studien nötig, um die Pathophysiologie und die Behandlungen für diese Haarbrüchigkeiten herauszufinden und um einen Vorhersagewert dafür bei HCA zu bestimmen.

## Resumo

**Contexto:**

A alopecia associada ao sequestro folicular (HCA, Alopecia X) é bem reconhecida em lulus da Pomerânia. Os autores desconhecem a existência de relatos de fragilidade pilosa em cães afetados.

**Hipótese/Objetivos:**

Após a observarmos a presença de anormalidades frequentes na haste pilosa de lulus da Pomerânia alopécicos, levantamos a hipótese de que os eventos de fragilidade pilosa seriam mais frequentes em cães com fenótipo de HCA quando comparados a cães com pelagem normal.

**Animais:**

Oito lulus da Pomerânia alopécicos ou cruzamentos com fenótipo de HCA e 36 lulus da Pomerânia não afetados com pelagem normal relatada pelo dono.

**Materiais e Métodos:**

Todos os cães afetados foram submetidos a exames dermatológicos e avaliações clinicopatológicas. Amostras de pelos, arrancadas de áreas afetadas ou obtidas por escovação de cães não afetados, foram examinadas microscopicamente para a presença de anormalidades estruturais. Eventos de fragilidade capilar foram caracterizados como tricorrexe nodosa (TN) ou divisões longitudinais e foram expressos por 10 mg de cabelo. Um intervalo de referência foi calculado a partir do número de eventos de fragilidade capilar nas amostras de cães não afetados.

**Resultados:**

O limite superior de referência (com 90% de confiança) de amostras de 35 lulus da Pomerânia não afetados (um *outlier* excluído) foi de 9,75 eventos de fragilidade capilar por 10 mg de pelo. A mediana (intervalo) de eventos de fragilidade em oito cães com um fenótipo de HCA foi de 66,0 (30,2–166,7) por 10 mg de cabelo.

**Conclusões e Relevância Clínica:**

Os clínicos devem realizar rotineiramente a tricografia em lulus da Pomerânia com HCA porque anormalidades capilares abundantes, particularmente a tricorrexe nodosa, podem contribuir para a má qualidade do pelo. Mais estudos são necessários para estabelecer a fisiopatologia e os tratamentos para esses eventos de fragilidade e para determinar seu valor preditivo para HCA.

## 摘要

**背景:**

博美犬的毛发周期阻滞性脱毛(HCA，X脱毛)已经被充分了解。作者未查到关于患犬毛发脆弱的报道。

**假设/目的:**

在观察到脱毛的博美犬经常出现毛干异常后，我们假设与具有正常皮毛的犬相比，具有HCA表型的犬的毛发脆弱会更频发。

**动物:**

8只脱毛的博美犬或具有HCA表型的杂交犬，以及36只健康博美犬，其主人报告毛发正常。

**材料和方法:**

所有患犬都接受了皮肤病检查和临床病理评估。从脱毛区域拔出或从健康犬身上刷牙获得的毛发样本在显微镜下进行了结构异常检查。头发脆弱的特征是结节性毛断裂(TN)或纵向裂开，每10毫克头发发生一次。根据健康犬的样本中毛发脆弱的数量计算参考区间。

**结果:**

35名健康博美犬(排除一个异常值)样本的参考上限(90%置信度)为每10毫克头发9.75次头发脆弱。8只具有HCA表型犬的脆性毛发中位数(范围)为每10毫克毛发66.0(30.2‐166.7)。

**结论和临床相关性:**

临床医生应定期对HCA博美患犬进行毛发检查，因为大量的毛发异常，特别是结节性毛发断裂，可能会导致毛发质量差。需要进一步的研究来确定这些脆性毛发的病理生理学和治疗方法，并确定其对HCA的预测价值。

## Résumé

**Contexte:**

L'alopécie associée à l'arrêt du cycle pilaire (HCA, Alopecia X) est bien connue chez les chiens de Poméranie. Les auteurs n'ont pas connaissance de rapports sur la fragilité des poils chez les chiens affectés.

**Hypothèse/Objectifs:**

Suite à l'observation d'anomalies fréquentes de la tige du poil chez les Poméraniens alopéciques, nous avons émis l'hypothèse que les événements de fragilité du poil seraient plus fréquents chez les chiens présentant un phénotype d'AHC par rapport aux chiens à pelage normal.

**Animaux:**

Huit chiens de Poméranie alopéciques ou croisés avec un phénotype HCA et 36 Poméraniens non affectés avec des pelages normaux selon le propriétaire.

**Matériels et méthodes:**

Tous les chiens atteints ont subi un examen dermatologique et des évaluations clinicopathologiques. Des échantillons de poils, prélevés sur les zones affectées ou obtenus par brossage sur des chiens non affectés, ont été examinés au microscope à la recherche d'anomalies structurelles. Les événements de fragilité des poils ont été caractérisés par une trichorrhexie noueuse (TN) ou des fentes longitudinales, et ont été exprimés pour 10 mg de poils. Un intervalle de référence a été calculé à partir du nombre d'événements de fragilité des poils dans les échantillons provenant de chiens non atteints.

**Résultats:**

La limite supérieure de référence (avec un niveau de confiance de 90 %) des échantillons de 35 Poméraniens non atteints (une valeur aberrante a été exclue) était de 9,75 événements de fragilité des poils par 10 mg de poils. La médiane (intervalle) des événements de fragilité chez huit chiens présentant un phénotype HCA était de 66,0 (30,2‐166,7) par 10 mg de poils.

**Conclusions et pertinence clinique:**

Les cliniciens devraient effectuer systématiquement une trichographie chez les Poméraniens atteints d'HCA, car des anomalies capillaires abondantes, en particulier le *trichorrhexis nodosa*, peuvent contribuer à la mauvaise qualité du poil. D'autres études sont nécessaires pour établir la pathophysiologie et les traitements de ces événements de fragilité et pour déterminer leur valeur prédictive pour l'HCA.

## 要約

**背景:**

毛周期停止を伴う脱毛症(HCA, Alopecia X)はポメラニアンでよく知られている。筆者らは罹患犬における毛の脆弱性の報告を知らない。

**仮説/目的:**

脱毛症のポメラニアンでは毛軸の異常が頻繁に観察されることから、HCAの表現型を持つ犬では、正常な被毛を持つ犬と比較して、毛の脆弱化現象がより頻繁に起こるという仮説を立てた。

**供試動物:**

HCAの表現型を持つ脱毛症のポメラニアンまたはその交配犬8頭、および飼い主が正常な被毛であると報告した非罹患ポメラニアン36頭。

**材料と方法:**

すべての罹患犬は皮膚科学的検査および臨床病理学的評価を受けた。罹患部位から摘出した毛サンプル、あるいは非罹患犬からブラッシングにより採取した毛サンプルを顕微鏡で観察し、構造的異常を調べた。毛の脆弱性事象は結節性裂毛症(TN)または縦裂として特徴づけられ、毛10mgあたりで表された。非罹患犬の毛脆弱事象の数から基準間隔を算出した。

**結果:**

35頭の非罹患ポメラニアン(1頭の異常値を除外)から得られた基準上限値(信頼度90%)は、毛髪10mgあたり9.75であった。HCAの表現型を有する8頭の犬における脆弱性事象の中央値(範囲)は、毛髪10mgあたり66.0(30.2‐166.7)であった。

**結論と臨床的意義:**

豊富な毛の異常、特に結節性裂毛症が毛質の低下に寄与している可能性があるため、臨床医はHCAを有するポメラニアンに毛検査をルーチンに実施すべきである。これらの脆弱性事象の病態生理学および治療法を確立し、HCAの予測値を決定するためにさらなる研究が必要である。

## RESUMEN

**Introducción:**

La alopecia asociada con el arresto del ciclo del pelo (HCA, Alopecia X) es bien conocida en los perros Pomeranian. Los autores desconocen informes de fragilidad del pelo en perros afectados.

**Hipótesis/Objetivos:**

Tras la observación de frecuentes anomalías del tallo del pelo en los Pomeranian alopécicos, planteamos la hipótesis de que los episodios de fragilidad del pelo serían más frecuentes en los perros con un fenotipo de HCA en comparación con los perros con pelaje normal.

**Animales:**

Ocho perros Pomeranian alopécicos o cruces con un fenotipo de HCA y 36 Pomeranian no afectados con pelaje normal según informaron los propietarios.

**Materiales y métodos:**

Todos los perros afectados se sometieron a un examen dermatológico y evaluaciones clinicopatológicas. Se examinaron microscópicamente muestras de pelo, arrancadas de las zonas afectadas u obtenidas mediante cepillado de perros no afectados, para detectar anomalías estructurales. Los eventos de fragilidad del cabello se caracterizaron como tricorrexis nodosa (TN) o hendiduras longitudinales, y se expresaron por cada 10 mg de cabello. Se calculó un intervalo de referencia a partir del número de eventos de fragilidad del cabello en las muestras de perros no afectados.

**Resultados:**

El límite superior de referencia (con un 90 % de confianza) de las muestras de 35 Pomeranian no afectados (se excluyó un caso atípico) fue de 9,75 eventos de fragilidad del cabello por cada 10 mg de cabello. La mediana (rango) de eventos de fragilidad en ocho perros con un fenotipo de HCA fue de 66,0 (30,2–166,7) por cada 10 mg de cabello.

**Conclusiones y relevancia clínica:**

Los clínicos deben realizar de forma rutinaria una tricografía en los Pomeranian con HCA porque las abundantes anomalías del cabello, en particular la tricorrexis nodosa, pueden contribuir a una mala calidad del pelaje. Se requieren más estudios para establecer la fisiopatología y los tratamientos para estos eventos de fragilidad y para determinar su valor predictivo para la HCA.

## INTRODUCTION

Hair shaft defects are rarely reported in veterinary dermatology, putatively because microscopical examination of hair shafts is not routinely performed as part of the diagnostic approach in animals with abnormal coats.^1^ This is unfortunate because structural hair abnormalities are easily diagnosed in hair specimens mounted for microscopy.^2^ Trichorrhexis nodosa (Greek: *tricho*, pertaining to the hair + *rrhexis*, rupture + Latin: *nodosa*, node‐like) (TN) is the most common hair shaft abnormality reported in human medicine; textbooks of hair diseases describe the typical light microscopical features as comprising a hair shaft fracture in which the individual cortical cells and their fragments splay out, resembling the ends of two brushes pushed into one another.^2^, ^3^

Trichorrhexis nodosa has been described in two Irish Water Spaniels with hypotrichosis/alopecia which resolved with nutritional management^4^ and an inherited form of TN was suspected in two unrelated Golden Retrievers.^5^ TN may be congenital or acquired and localised or generalised. The acquired form is often caused by chemical and physical trauma to the hair shaft. Both the bald thigh syndrome of Greyhounds^6^ and follicular dysplasia in Curly‐coated Retrievers^7^ are associated with transverse fractures and other structural defects of the hair shaft and not with TN. Trichoptilosis, a longitudinal splitting of the hair shaft at its distal end (‘frizzies’ and ‘split ends’), is reported in both humans and dogs.^1^

The Pomeranian dog breed has been recognised since the 1970s as being predisposed to an alopecic skin disorder characterised by bilaterally symmetrical alopecia and hyperpigmentation that primarily affects male dogs of <4 years of age.^8^, ^9^, ^10^ Absence of a clear understanding of the pathogenesis underpins the previous use of various terms such as pseudo‐Cushing's syndrome,^10^ growth hormone‐responsive alopecia in the mature dog (to differentiate it from pituitary dwarfism),^9^ hyposomatotropism in the mature dog,^8^ congenital adrenal hyperplasia‐like syndrome^11^, ^12^ and Alopecia X.^13^ More recently, the presentation has been more usefully referred to as hair cycle arrest (HCA)^14^, ^15^ which reflects a predominant histopathological feature in the context of the hair growth cycle. The term black skin disease^14^ is in common usage among Pomeranian owners in the United Kingdom.^16^

Before study conception, we observed frequent hair shaft abnormalities, primarily of the TN type, in eight Pomeranian‐type dogs that were presented consecutively with alopecia typical of the phenotype of HCA in that breed. We considered that this feature may contribute to and exacerbate the abnormal coat appearance in affected dogs. We hypothesised that quantitative study would show that fragility events were abundant in Pomeranian dogs with the phenotype of HCA and much less frequent in Pomeranians with normal hair coats.

## MATERIALS AND METHODS

### Enrolment and ethics

Affected dogs comprised Pomeranians or their crosses that were referred to Dermatology Service of the Queen Mother Hospital for Animals (QMHA), Royal Veterinary College (RVC), Hatfield or to Bayswater Referral Clinic (BRC), London, for investigation of progressive symmetrical alopecia. The unaffected Pomeranians were enrolled at a dog show (The Pomeranian Club UK 2023 Championship show). Permission for study participation was obtained by written informed consent from dog owners under study approval by RVC's Clinical Research Ethical Review Board [URN M2022‐0172] and Social Sciences Ethical Review Board [URN SR2022‐147]. Routine clinical evaluation of the alopecic dogs was done with informed owner consent under the auspices of the UK Veterinary Surgeons Act.

### Clinical assessments

#### Affected dogs

For affected dogs referred for assessment of alopecia, historical information was obtained from previous and referring veterinary surgeons and from the owners at the time of consultation. Each dog underwent a general physical and dermatological examination. Dogs were recruited to the study with clinical signs consistent with the HCA phenotype of Pomeranian dogs and concurrent abundant hair fragility events on microscopical examination of hair samples. Phenotypic features of HCA comprised bilaterally symmetrical, nonpruritic, grossly noninflammatory alopecia and sometimes hyperpigmentation that developed in dogs of <4 years of age, with sparing of the head and distal limbs. Dogs were otherwise healthy Pomeranian or Pomeranian crosses without evidence of infestation and infection on microscopical examination of hair plucks. Dogs in which clinicopathological or histopathological testing was performed were excluded if those tests contradicted the diagnosis of HCA (e.g. inconsistent histopathological findings and evidence of hypothyroidism).^8^, ^9^, ^10^ No attempt was made to standardise laboratory assessment. Tests performed included haematological and serum biochemical investigation, urinalysis, fungal cultures, microscopy for parasites, tests of thyroid and adrenal function and skin biopsy analysis.

#### Unaffected dogs

The owners of 36 adult Pomeranian dogs with reportedly normal coats attending the Championship Show provided details of their dogs' age and sex and were asked to record their observations on the quality of their dog's hair coat (Table S1 in Supporting Information). No veterinary evaluation was performed on these animals.

### Hair sampling

#### Affected dogs

Hair samples were collected using sterile haemostats (without protective tubing) and traction as part of the routine investigation into alopecia in affected dogs. Between two and six plucks were obtained from areas of abnormal hair coat on the neck or trunk, each typically yielding 70–110 hairs. Hairs were mounted in liquid paraffin and examined using a light microscope for evidence of ectoparasitism, fungal invasion of hair shafts or presence of arthrospores and structural abnormalities of the hair shafts.

#### Unaffected dogs

Dogs attending the championship show were groomed using brushes before entering the show ring by their owners. Hair was collected from the brushes, most often by the use of a comb and placed in a labelled paper envelope for subsequent examination. This method typically yielded hair samples of 40–190 mg.

### Quantification of structural abnormalities of hair shafts in healthy and affected dogs

It was established that a 6‐ to 10‐mg sample of hair, mounted in 0.65 mL of liquid paraffin, filled the area beneath a rectangular glass coverslip (22 × 50 mm) with adequate dispersal of the hairs so that individual shafts could be examined using the ×4 scanning objective of a light microscope (×40 magnification). Hair samples were weighed (AC210S Analytical Balance; Sartorius AG) in plastic Petri dishes of 60 × 15 mm diameter (Falcon Easy Grip Tissue Culture Dish, 3004; Thermo Fisher Scientific), with a target weight of 7–10 mg.

**FIGURE 1:**
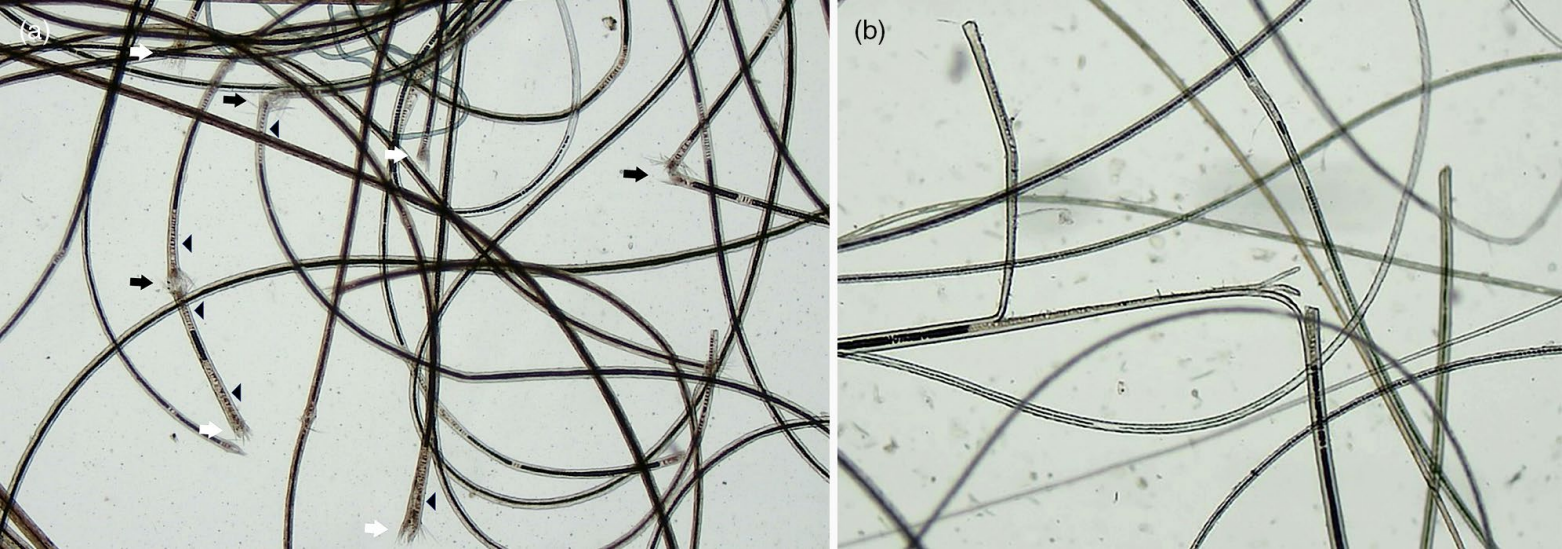
(a) Trichorrhexis nodosa (TN) hair fragility events in a Pomeranian dog with the phenotype of hair cycle arrest (HCA). Fractured hair shaft with splayed fragments resembling the ends of two brushes pushed together yet without separation of the ends (‘TN shaft’, black arrows) and fractured ends of hair shaft with splayed fragments resembling a brush (‘TN end’, white arrows). Each TN event is associated with an adjacent irregularity of medullary pigmentation (arrowheads). (b) Longitudinal split resembling a ‘greenstick’ fracture in a primary hair from a Pomeranian dog with a phenotype of HCA (‘LS shaft’).

Hairs mounted in liquid paraffin were examined using a light microscope with ×40 magnification. The image was displayed on a television monitor. Each specimen was systematically examined in its entirety by covering the area beneath the coverslip in a tramline method. The microscope images were examined by a panel of at least four veterinarians (EB, JJ, AL and RB). Structural hair abnormalities were recorded as ‘TN end’ (TN; fractured end of hair shaft with splayed fragments resembling a brush) (Figure 1a), ‘TN shaft’ (TN; hair shaft fracture in which the individual cortical cells and their fragments splay out, resembling the ends of two brushes pushed into one another yet without separation of the ends) (Figure 1a), ‘LS end’ (longitudinal split end of a fractured hair shaft, with or without splayed fragments resembling a brush) and ‘LS shaft’ (longitudinal splits within a hair shaft without transverse fracture) (Figure 1b).

A method was devised to quantify hair structure abnormalities in each specimen, expressed as total number of fragility events per 10 mg of hair, according to the formula:

$$\text{Total fragility events}\;\mathrm{per}\;10\;\mathrm{mg}=\left[\left(0.5^{*}\mathrm{TN}\;\mathrm{end}\right)+\mathrm{TN}\;\text{shaft}+\left(0.5^{*}\mathrm{LS}\;\mathrm{end}\right)+\mathrm{LS}\;\text{shaft}\right]*10/W,$$

where W is the weight of hair applied to the slide in mg. TN ends and LS ends were each regarded as half (0.5) of a fragility event because one TN or LS shaft event will generate two ends in the event of progression to complete hair fracture. The observations were repeated on the same slide collection on a separate occasion by the same investigators (AL and RB) using a multi‐headed Nikon microscope; the mean value obtained for each sample was used in subsequent analyses. Observers were unaware of the status (affected versus unaffected dog) of the specimen in each case.

### Statistical methods

A reference interval was determined from hair fragility values (events per 10 mg of hair) in the unaffected dogs; outlying values were excluded by Dixon's method.^18^ Following ASVCP guidelines^18^ and a nonparametric technique,^19^ the 90% confidence interval (CI) was defined by the 10th and 90th percentiles inclusive, with the upper limit set as the 90th percentile plus 1.5× the interquartile range. The lower limit was defined as 0, corresponding to absence of hair fragility events.

## RESULTS

### Signalment of dogs presenting with HCA phenotype

Eight dogs were enrolled, comprising seven Pomeranians and one Pomerian X Siberian husky. The mean (±SD) age at onset of coat abnormalities was 19.6 (±7.5) months (range 12–34 months) (Table 1). The mean (±SD) age at presentation was 33 (±12.8) months (range 19–55 months). The mean (±SD) duration of signs before presentation was 13.4 (±7.1) months (range 3–24 months). Seven dogs were male (three entire) and one was a neutered female. Four dogs were white/cream, two were orange and two were sable.

**TABLE 1:** Signalment, age at onset and duration of alopecia in eight Pomeranian‐type dogs with hair fragility and a phenotype of hair cycle arrest.

Dog no.	Breed	Sex	Age at onset	Age at presentation	Duration
AP9	Pomeranian	FN	2 y 10 m	4 y 7 m	21 m
AP10	Pomeranian	M	1 y 3 m	2 y	9 m
AP11	Pomeranian × Husky	M	1 y 9 m	2 y 10 m	13 m
AP13	Pomeranian	M	12 m	2 y 6 m	18 m
AP14	Pomeranian	MN	2 y	4 y	24 m
AP15	Pomeranian	MN^a^	16 m	1 y 7m	3 m
AP16	Pomeranian	MN	1 y 11 m	2 y 9 m	10 m
AP17	Pomeranian	MN	12 m	1 y 9 m	9 m

Abbreviations: FN, neutered female; M, male; MN, neutered male; m, months; y, years.

^a^Neutered during investigation.

### Clinical signs in dogs presenting with HCA phenotype

**FIGURE 2:**
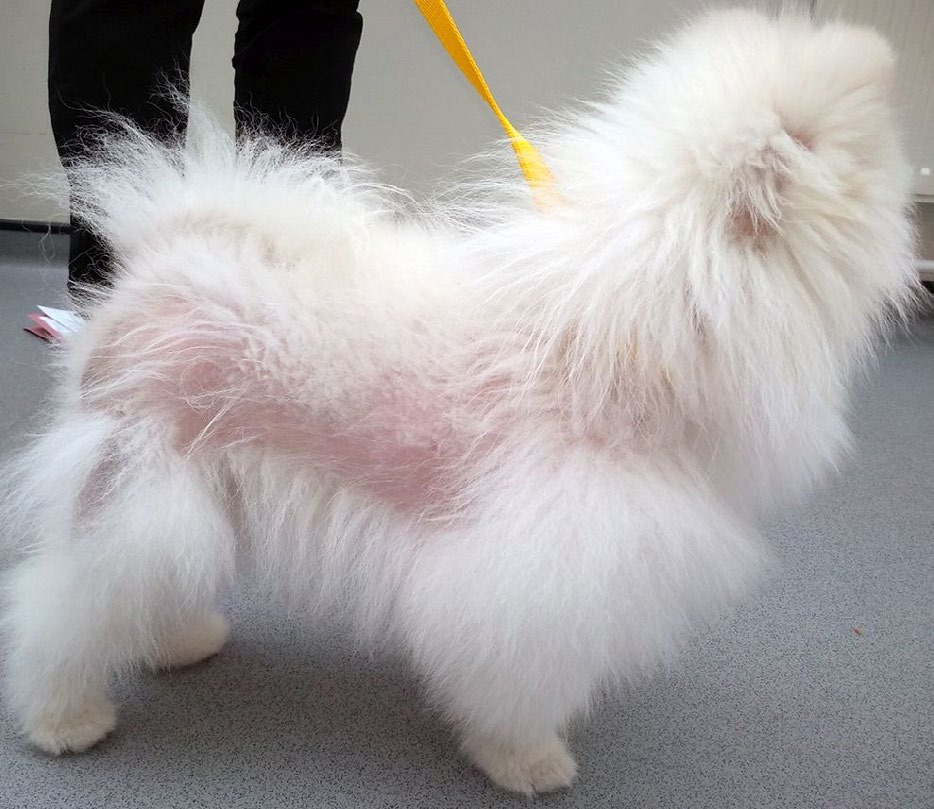
Truncal alopecia in a Pomeranian dog with hair cycle arrest and trichorrhexis nodosa.

Hair coat changes commonly preceded alopecia; reported changes included the development of a ‘woolly’ (*n* = 5) or ‘short and fluffy’ (*n* = 1), generally ‘brittle, poor quality’ (*n* = 1) and ‘soft and frizzy’ (*n* = 1) hair coat that were often prone to matting. This progressed to symmetrical alopecia in all dogs which involved the trunk yet consistently spared the head and distal limbs (Figure 2). Hyperpigmentation of the alopecic skin was evident in four of eight cases. Other findings reported or identified included erythematoceruminous otitis externa in one dog and paraphimosis in another. One dog had a history of a fractured radius and widespread superficial pyoderma; the remaining dogs had no concurrent diseases.

### Laboratory investigations in dogs presenting with HCA phenotype

Microscopical examination of skin and coat samples failed to identify the presence of ectoparasites in each case (hair plucks, *n* = 8; coat brushings, *n* = 4; skin scrapings, *n* = 2). Pathogenic fungi were not isolated from any of the six dogs from which samples were submitted for fungal culture and arthrospores were not observed in hair plucks from any the eight dogs.

Complete blood counts were unremarkable in all six dogs that were sampled (Table 2). Serum biochemical analysis was not performed in two dogs, was unremarkable in four, and two dogs had a mild increase in serum alkaline phosphatase (225 and 290 U/L; reference range <200 U/L). Two dogs were considered euthyroid based on measurements of both total thyroxine (T4) and thyroid‐stimulating hormone (TSH) (Table 2). Five dogs had normal basal total T4 values and one was not tested. Two dogs (dog AP 12 and AP 16) of three tested by adrenocorticotropic hormone (ACTH) stimulation showed elevated concentrations of cortisol or 17‐hydroxyprogesterone (Table 3). Results of these ACTH stimulation tests are consistent with previous reports in both healthy Pomeranians and dogs with HCA.^20^, ^21^

**TABLE 2:** Results of laboratory tests and fragility counts in eight Pomeranian‐type dogs with hair fragility and a phenotype of hair cycle arrest. Normal ranges are given in parentheses.

Dog no.	CBC	Blood biochemical result	Total T4 (10–55 nmol/L)	Free T4 (6–40 pmol/L)	TSH (<0.41 ng/mL)	Histopathological findings	Hair fragility^a^	Brushing habits
AP9	NSA	ALP 225 U/L (38–200)	44.4	ND	ND	ND	56.0	Weekly
AP10	NSA	NSA	22.3	ND	ND	ND	54.3	Daily
AP11	ND	NSA	26	ND	ND	ND	71.1	Avoided
AP13	NSA	NSA	35	ND	ND	ND	94.8	Frequency NR
AP14	ND	ND	ND	ND	ND	ND	61.0	No record
AP15	NSA	ND	20.8	14.6	0.09	ND	166.7	No record
AP16	NSA	NSA	22	ND	ND	Atrophic pattern	76.1	No record
AP17	NSA	ALP 290 U/L(38–200)	14.7	ND	0.03	Atrophic pattern	30.2	Every other day

Abbreviations: ALP, alkaline phosphatase; CBC, complete blood count; n, normal range; ND, not done; NQ, not quantified; NR, not recorded; NSA, no significant abnormality; T4, thyroxine; TSH, thyroid‐stimulating hormone; WNL, reported as within normal limits although value not available.

^a^ Hair fragility expressed as number of fragility events (trichorrhexis nodosa and longitudinal splits) per 10 mg of hair.

**TABLE 3:** Concentrations of cortisol and 17‐hydroxyprogesterone before (pre) and after (post) adrenocorticotrophic hormone stimulation in three alopecic Pomeranian dogs with a phenotype of hair cycle arrest and concurrent trichorrhexis nodosa.

Dog no.	Cortisol pre (RI 28–250 nmol/L)	Cortisol post (RI 200–600 nmol/L)	17‐OHP pre (RI <3.0 nmol/L)	17‐OHP post (RI 3.0–8.0 nmol/L)
AP 12	102	352	4.3^a^	8.1^a^
AP 15	105	137	ND	ND
AP 16	47	909^a^	1.74	3.34

Abbreviations: ND, not done; 17‐OHP, 17‐hydroxyprogesterone; RI, reference interval.

^a^ Exceeds reference interval.

Histopathological examination of skin biopsy specimens in two cases (Table 2) showed features consistent with HCA of the Pomeranian dog.^13^, ^23^, ^24^ Both dogs had mild orthokeratotic hyperkeratosis and mild epidermal melanosis. Hair follicles were in the telogen phase, with mild follicular keratosis and in one case, several follicles showed excessive trichilemmal keratinisation (‘flame follicles’). The dermis was subjectively thinned in one case and inflammation was not observed in sections from either dog.

### Treatment of Pomeranian dogs presenting with an HCA phenotype

Six dogs were receiving broad‐spectrum ectoparasiticides for routine prevention (oral isoxazoline, *n* = 4; spot‐on imidacloprid‐containing product, *n* = 1; spot‐on fipronil, *n* = 1).

Four dogs had received melatonin (0.9–4 mg/kg/day) before referral for 2–9 months; one dog had shown partial hair regrowth. Four dogs had not received specific treatment for HCA before referral. Ancillary treatments comprised topical products (spot‐on essential fatty acid products [*n* = 1]; emollients [*n* = 2]) or salmon‐oil supplementation ([*n* = 1]).

The grooming habits of the owners was mentioned in the records of five cases (Table 2). Where a frequency was specifically recorded it was daily or every other day and one owner had purposefully avoided brushing since the onset of alopecia.

### Hair samples from Pomeranian dogs presenting with an HCA phenotype

Hair fragility events were abundant in alopecic dogs. TN was the most frequent abnormality; this resulted in many fragile and broken shafts, sometimes with several events in the one hair (Figure 1), while LS and trichoptilosis (‘greenstick fractures’) (Figure 2) were less frequent. TN events were often associated with an irregular loss of medullary pigmentation in adjacent portions of the hair shaft (Figure 1). TN events exceeded LS events by approximately 17:1. The median (range) number of hair fragility events per 10 mg of hair in affected dogs was 66.0 (30.2–166.7).

### Hair samples from unaffected Pomeranian dogs

Of the 36 healthy dogs sampled at the show, 24 were orange or orange‐sable, 6 were wolf sable, 2 were cream or shaded sable, 3 were black and 1 was red. All 36 of these dogs were reported by their owners to have good coat condition with no hair loss. The mean (±SD) age at sampling of 33 of the 36 healthy dogs (three missing values) was 42 (±30) months (range 10–166 months).

**FIGURE 3:**
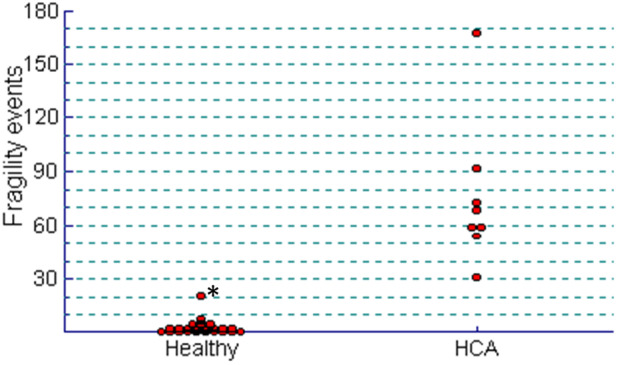
Dot plot illustrating the total number of hair fragility events (trichorrhexis nodosa and longitudinal splits) observed by light microscopy and expressed per 10 mg of hair in 36 healthy Pomeranian dogs with abnormal hair coat and eight dogs with a phenotype of hair cycle arrest of the Pomeranian. Value highlighted by * is identified as an outlier (D/R = 0.59 by Dixon's method).

The median (upper, lower quartile) number of hair fragility events per 10 mg of hair was 2.3 (0.8, 3.9), with a range of 0.3–20.2 (Figure 3). A value of 20.2 was identified as an outlier (D/R = 0.59, *n* < 0.3)^18^ and omitted from further analyses. With the outlying value excluded, the median (upper, lower quartile) number of hair fragility events per 10 mg of hair was 2.3 (0.8, 3.9), with a range of 0.3–8.2. The 90% one‐tailed robust reference interval was 0–9.75 hair fragility events per 10 mg of hair. TN events exceeded LS events by approximately 3:1.

The relationship of the individual hair fragility values in unaffected Pomeranian dogs and dogs with an HCA phenotype is illustrated in Figure 3.

## DISCUSSION

This study demonstrates that hair shaft abnormalities, primarily of the TN type, may coexist with classical alopecic signs of HCA in Pomeranian dogs. We are unaware of previous reports of hair fragility events in this breed, which is likely to be in accordance with previous reports of infrequent systematic hair shaft examination in dogs presenting with alopecia.^1^ The low frequency of hair fragility in the healthy show dogs highlights the marked difference between healthy Pomeranians and those with an HCA phenotype. It is difficult to assess the relative contribution of hair fragility and cycle arrest to the alopecia in the affected dogs, yet our microscopic findings fit well with frequent owner observations of poor coat quality. In future studies, there may be merit in assessing hair fragility in unaffected areas of alopecic dogs.

In human medicine the term ‘weathering’ is applied to all types of cosmetic manipulation that causes physical or chemical trauma to the hair shaft that may increase its fragility.^3^ Weathering may be relevant in the context of plush‐coated dogs, such as Pomeranians, that are frequently and enthusiastically groomed by their owners. Trauma alone, however, is most unlikely to account for the TN seen in the dogs with HCA, considering that the show Pomeranian dogs (subjected to vigorous grooming) had very few hair fragility events.

It is intuitive that the hair fragility observed in the affected dogs reflects abnormalities in keratins or other structural proteins necessary for hair integrity. The fragility may co‐occur with HCA in the absence of a direct mechanistic relationship between these abnormalities or may represent a key abnormality with HCA as a final outcome; both scenarios fit well with recent studies of transcriptome profiles in Pomeranian dogs with HCA that highlighted multiple examples of differential expression of genes involved in cell differentiation in the hair matrix, in addition to genes involved in anagen induction and promotion.^17^ If the hair fragility events reported here are confirmed to be closely associated with this HCA phenotype, their observation may guide further work into its pathogenesis.

Trichorrhexis nodosa in children is often associated with metabolic disorders (such as argininosuccinic aciduria) or forms of congenital ectodermal dysplasia. The affected Pomeranian dogs did not show signs of systemic disease, or the involvement of other ectodermal tissues (such as tooth and nail defects) associated with these syndromes.^3^, ^23^ The wide range of coat colours represented in the affected dogs does not support a close association between TN and hair pigmentation, even although focal medullary defects in pigmentation were commonly observed adjacent to TN events in affected hairs.

A limitation of the study is that the diagnostic evaluations of the alopecic Pomeranian dogs did not include a standardised set of diagnostic tests. We are not aware of uniformly accepted diagnostic criteria for HCA in Pomeranian dogs. The clinical diagnosis is based on compatible signalment, history, clinical signs and exclusion of other causes of alopecia, especially endocrine diseases such as hyperadrenocorticism and hypothyroidism.

Dynamic tests of adrenal function were performed in a small number of dogs. In no case did the clinical presentation support hyperadrenocorticism as a final diagnosis although some affected dogs had abnormal steroidogenesis.^20^, ^21^, ^22^ We are not aware of reports of TN in direct association with disorders of thyroidal or pituitary/adrenal function.

A further limitation is that the show Pomeranian dogs were not subjected to veterinary examination. The owners reported their own perception of hair coat quality. Hair samples were obtained from the affected dogs using haemostats, while healthy show Pomeranians were sampled by brushing. This is not problematic because hair plucking by haemostats produces only rare defects of a morphology that is distinct from TN. We conducted an unpublished study to confirm our experience that hair fragility events of the TN type do not occur as an artefact of sampling by this method (data available on request). In addition, our examinations of the hair samples collected by brushing from healthy dogs indicate that the technique is not traumatic to hair shafts, even in the face of the vigorous grooming that was practiced at a national championship dog show.

A concern for breeders of Pomeranian dogs is that some young dogs enter the breeding pool before HCA becomes evident. Coat texture changes were noted in advance of alopecia in some cases and further studies are required to determine whether these texture changes are associated with fragility events and whether these hair coat abnormalities may herald the onset of HCA. If confirmed, screening for hair fragility might inform on the advisability of future breeding from individual dogs.

## CONCLUSIONS

Clinicians should routinely perform trichography in alopecic Pomeranian dogs with HCA because hair shaft abnormalities, particularly TN, may contribute to poor coat quality. Further studies are required to establish the pathophysiology and prognostic value of, and to devise treatments for, these fragility events.

## AUTHOR CONTRIBUTIONS

**Erin Brennan:** Conceptualization; data curation; formal analysis; investigation; methodology; resources; visualization; writing – original draft. **Jonna Juhola:** Investigation; methodology; resources; writing – review and editing. **Ewan A. Ferguson:** Investigation; resources; supervision; writing – review and editing. **Anette Loeffler:** Investigation; methodology; resources; supervision; writing – review and editing. **Rosario Cerundolo:** Investigation; resources; writing – review and editing. **Anke Hendricks:** Investigation; resources; visualization; supervision; writing – review and editing. **Ross Bond:** Conceptualization; data curation; formal analysis; investigation; methodology; project administration; resources; supervision; visualization; writing – original draft.

## FUNDING INFORMATION

Self‐funded.

## CONFLICT OF INTEREST STATEMENT

The authors have no conflict of interest to declare.

## Supporting information


Table S1.
