# Supplementary material for: Hair fragility (trichorrhexis nodosa) in alopecic Pomeranian dogs
Source: Vet Dermatol. 2024 Sep 30;36(1):64–73. doi: 10.1111/vde.13296 (PMC11696478; doi:10.1111/vde.13296)
Supplement: Supplementary file 1 — Table S1. [file VDE-36-64-s001.docx]

**Table S1**. Questionnaire recording owner observations on the hair coat quality of Pomeranian dogs attending a championship dog show.

| Name |  |
| --- | --- |
| Age |  |
| Sex (select one) | M entire, M neutered, F entire, F neutered |
| Coat colour  (select one) | Orange, red, white, black, merle, wolf sable, chocolate, blue, cream, brindle, black and tan, beaver, tricoloured. |
| Coat status  (select one) | 1. Always good coat and no abnormal hair loss 2. Current or previous hair loss without frizzy coat 3. Current or previous hair loss without frizzy coat |
| Coat quality  (select one if applicable) | 1. If frizzy, since puppy? 2. If frizzy, developed later in life? |
| Other observations on haircoat |  |
